# ESR Essentials: Perfusion MRI—practice recommendations by the European Society for Magnetic Resonance in Medicine and Biology

**DOI:** 10.1007/s00330-025-12306-5

**Published:** 2026-01-27

**Authors:** Petra J. van Houdt, Lena Václavů, Steven Sourbron, Eve S. Shalom, Christian Federau, Mami Iima, Mira M. Liu, Linda Knutsson, Ronnie Wirestam, Matthias Günther, Matthias J. P. van Osch, Rianne A. van der Heijden

**Affiliations:** 1 Department of Radiation Oncology, The Netherlands Cancer Institute, Amsterdam, The Netherlands; 2 C.J. Gorter MRI Center, Department of Radiology, Leiden University Medical Center, Leiden, The Netherlands; 3 Division of Clinical Medicine, University of Sheffield, Sheffield, UK; 4 AI Medical AG, Zollikon, Switzerland; 5 University of Zürich, Zürich, Switzerland; 6 Department of Fundamental Development for Advanced Low Invasive Diagnostic Imaging, Nagoya University Graduate School of Medicine, Nagoya, Japan; 7 BioMedical Engineering and Imaging Institute, Icahn School of Medicine at Mount Sinai, New York, NY USA; 8 F.M. Kirby Research Center for Functional Brain Imaging, Kennedy Krieger Institute, Baltimore, MD USA; 9 Department of Neurology, Johns Hopkins University School of Medicine, Baltimore, MD USA; 10 Department of Medical Radiation Physics, Lund University, Lund, Sweden; 11 Fraunhofer Institute for Digital Medicine MEVIS, MR Physics, Bremen, Germany; 12 MR-Imaging and Spectroscopy, University of Bremen, Bremen, Germany; 13 mediri GmbH, Heidelberg, Germany; 14 Leiden Institute of Brain and Cognition (LIBC), Leiden University, Leiden, The Netherlands; 15 Department of Radiology & Nuclear Medicine Erasmus University Medical Center, Rotterdam, The Netherlands; 16 Department of Radiology, University of Wisconsin-Madison, Madison, WI USA

**Keywords:** Perfusion magnetic resonance imaging, Contrast media, Radiologists

## Abstract

**Abstract:**

Perfusion MRI techniques—including dynamic susceptibility contrast (DSC) MRI, dynamic contrast-enhanced (DCE) MRI, arterial spin labeling (ASL) MRI, and intravoxel incoherent motion (IVIM) MRI—hold strong potential as imaging techniques for diagnosing, staging, and monitoring disease across a range of clinical applications. However, clinical adoption, especially of quantitative parameters, remains variable across techniques. Key barriers to broader implementation include a lack of standardized acquisition and analysis protocols, leading to poor reproducibility and reduced clinical confidence. Additionally, limited awareness and understanding of certain techniques among radiologists contribute to underutilization in practice. This work provides practice recommendations to support radiologists in integrating perfusion MRI into routine clinical workflows. It includes guidance on technique selection, acquisition, and analysis, supported by a flowchart outlining typical imaging pathways. These efforts align with ongoing initiatives such as the Quantitative Medical Imaging Coalition (formerly QIBA) and the ISMRM Open Science Initiative for Perfusion Imaging (OSIPI), which are developing standards and tools to enhance reproducibility and clinical utility. Ultimately, the successful adoption of state-of-the-art perfusion MRI depends on close collaboration between clinicians, researchers, and industry stakeholders to ensure robust, standardized, and clinically meaningful application.

**Key Points:**

- *Perfusion MRI parameters hold great promise as imaging biomarkers, but their clinical adoption, especially of quantitative parameters, remains variable across perfusion MRI techniques*.
- *An overview of perfusion MRI techniques, explaining the physics, illustrating clinical applications, and addressing common technical challenges, is provided to support perfusion MRI use in clinical practice*.
- *Successful adoption of state-of-the-art perfusion MRI depends on close collaboration between clinicians, researchers, and industry stakeholders to ensure robust, standardized, and clinically meaningful applications for patient care*.

## Key Recommendations

- Dynamic susceptibility contrast MRI protocols (2D gradient echo with echo planar imaging (EPI) readout) should provide whole-brain coverage, capture the contrast agent bolus’ shape (temporal resolution 1–1.5 s) and provide sufficient signal-to-noise ratio (SNR): contrast agent dose (0.1 mmol/kg), TE (1.5 T: 35–45 ms, 3 T: 25–35 ms) and flip angle (60–70°). In case of blood-brain-barrier disruption, correction for leakage is needed (Technical efficacy stage 4 [1]).
- Dynamic contrast-enhanced MRI protocols (3D-spoiled gradient recalled echo) need a careful trade-off between temporal and spatial resolution/coverage, and SNR, depending on the clinical application. A baseline T1 map (plus B1 map for ≥ 3 T) is needed for quantitative analysis, allowing the conversion of signal intensity to contrast agent concentration (Stage 2).
- For arterial spin labeling (pseudocontinuous arterial spin labeling with segmented 3D readout), the post-label-delay time should be long enough for spins to travel to the tissue, but short enough to avoid signal loss due to T1 decay. Single-delay ASL is widely available for clinical use (Stage 5), whereas multi-delay ASL can provide additional arrival-time maps (Stage 3).
- Intravoxel incoherent motion protocols (single-shot EPI) should include ≥ 3 directions, a b-value set with values in both the pseudo-diffusion (0–200 s/mm^2^) and diffusion regime (200–1000 s/mm^2^) based on the organ of interest, motion correction and averaging for increased SNR (Stage 2).

## Introduction

Perfusion ensures the constant delivery of oxygen and nutrients via the bloodstream to tissues, thus serving as a physiological indicator of tissue function. Perfusion MRI techniques enable the assessment of tissue vascularity and microcirculation, providing critical information for diagnosing, staging, and monitoring various diseases. The four principal MRI techniques are: Dynamic Susceptibility Contrast (DSC) MRI, Dynamic Contrast-Enhanced (DCE) MRI, Arterial Spin Labeling (ASL) MRI, and Intravoxel Incoherent Motion (IVIM) MRI. DSC-MRI and DCE-MRI require intravenous contrast agent administration, whereas ASL-MRI and IVIM-MRI are non-contrast techniques. Each method utilizes unique physical principles, enabling tailored applications in diverse clinical scenarios.

Despite their potential, each technique presents certain challenges, including susceptibility to artifacts, complex postprocessing requirements, need for substantial on-site technical expertise, and technique-specific limitations. In current clinical practice, perfusion MRI is often limited to visual assessment or semiquantitative analysis. Although quantitative perfusion MR parameters hold potential to serve as robust imaging biomarkers—providing objective thresholds for diagnosis and response monitoring—their clinical use remains limited.

This work provides an overview of perfusion MRI techniques, explaining the physics, illustrating key clinical applications, and addressing common technical challenges. The goal is to support radiologists in integrating state-of-the-art perfusion MRI into clinical practice.

## Perfusion MRI

### Dynamic susceptibility contrast (DSC) MRI

#### Basic physics

**Fig. 1:**
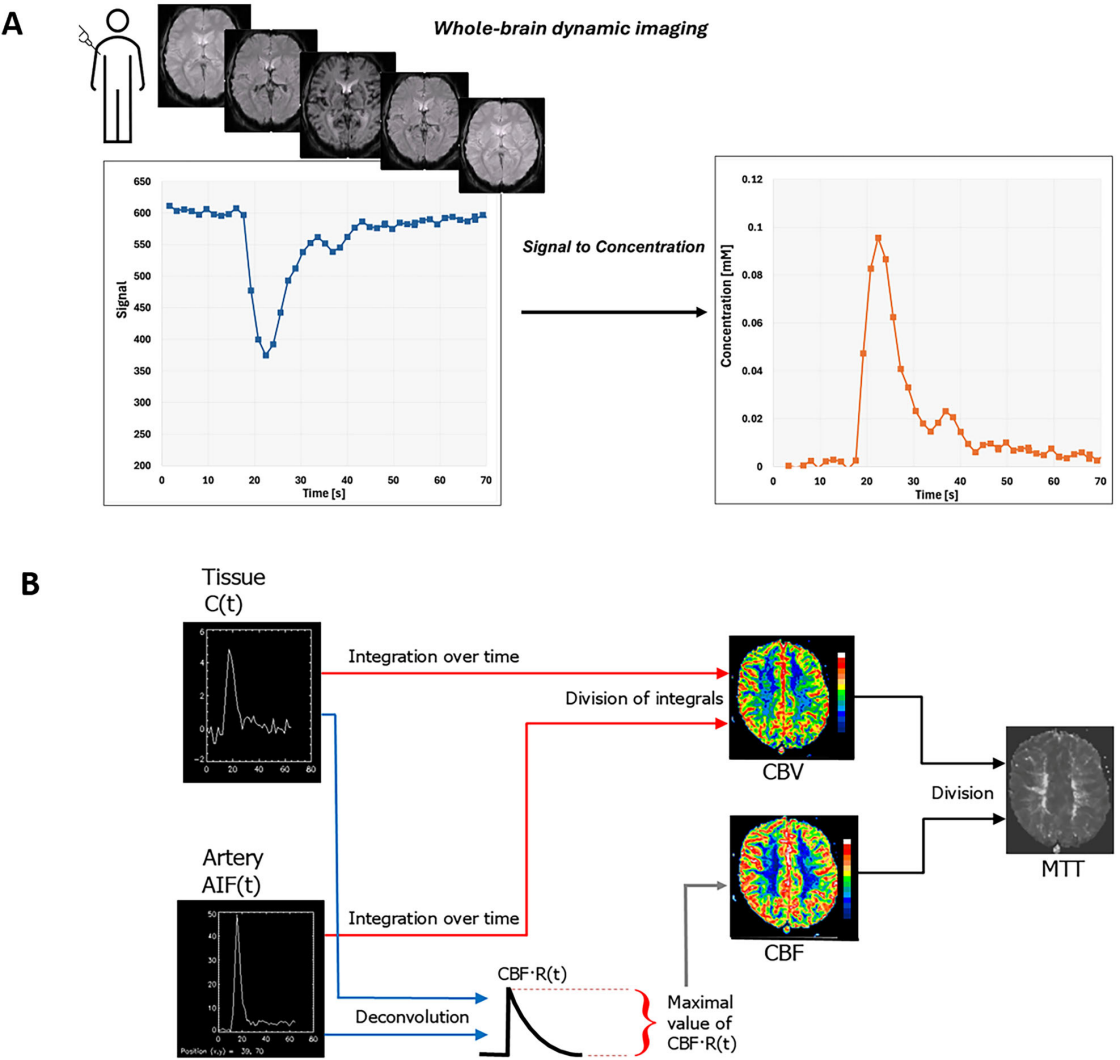
**A** Schematic overview of the dynamic susceptibility contrast (DSC-)MRI methodology from contrast agent (CA) injection to the calculation of the concentration-time curve in each voxel. After CA injection in an arm vein, the CA bolus will cause a temporary signal decrease when it passes through the brain tissue. The CA produces local magnetic field gradients that extend from the vascular compartment into the surrounding tissue. This creates a distribution of local resonance frequencies leading to phase dispersion of the water proton spins and a corresponding signal decrease. Before the quantification process, the signal-time curve of each voxel is converted into a concentration-time curve. **B** Schematic overview of the quantification procedure for obtaining cerebral blood volume (CBV), cerebral blood flow (CBF) and mean transit time (MTT). After conversion of signal to concentration C, the tissue and arterial concentration curves are integrated over time, and CBV is given by the tissue time integral divided by the arterial input function (AIF) time integral. CBF is given by the maximal value of the function obtained by deconvolution of the tissue concentration curve with the AIF. MTT is given by the CBV-to-CBF ratio

In DSC-MRI, tracking of a contrast agent (CA) bolus passage in tissue and artery is accomplished by rapid T2- or T2*-weighted MRI, in combination with general tracer kinetic theory, to obtain estimates of cerebral blood flow (CBF) and other perfusion-related parameters [2, 3]. The intravascular CA causes magnetic field inhomogeneities, leading to transverse relaxation time shortening and corresponding signal decrease (Fig. 1A).

The signal-time curve is used to obtain semiquantitative parameters such as bolus arrival time (BAT) and time-to-peak (TTP). However, these parameters’ dependence on both protocol settings and cardiovascular output limits their ability to accurately reflect the underlying physiology. To improve specificity, quantitative analysis is often preferred, including conversion of signal-time curves to concentration-time curves and registration of an arterial CA concentration curve, also known as the arterial input function (AIF). Figure 1B gives an overview of the quantification procedure for CBF, cerebral blood volume (CBV) and mean transit time (MTT). Table 1 summarizes general protocol recommendations, including adaptations to different pathologies. Other recommendations, primarily related to assessment of relative CBV changes in gliomas, are given by the Quantitative Imaging Biomarker Alliance (QIBA) DSC-MRI biomarker committee [4].

**Table 1:** Recommended protocol for DSC-MRI measurements in the brain

Parameter	Recommendation
Experimental setup	
Contrast agent dose	Single dose (0.1 mmol/kg)
Saline flush	20–30 mL
Injection rate	3–5 mL/s
IV catheter gauge	20 gauge (range 22 to 14 gauge)
Pulse sequence	2D GRE
Readout	EPI
Flip angle	60–70°
TE	Single echo: 35–45 ms at 1.5 T; 25–35 ms at 3 T Multi echo: 15/35–45 ms for AIF/Tissue at 1.5 T 12/25–35 ms for AIF/Tissue at 3 T
Temporal resolution	1–1.5 s
Matrix	128 × 128
FOV	220 × 220 (range 200 × 200 to 260 × 260)
Slice thickness	3–5 mm
No. baseline dynamics	30
Scan duration	At least 2 min
For pathologies with blood-brain barrier disruption, the following corrections should be applied, either alone or in combination.	
Prebolus/preload	0.025–0.1 mmol/kg, at least 5 min before DSC-MRI; Reduces mainly T1-induced signal contamination
Mathematical correction	Reduces both T1- and T2*- induced contaminations
Flip angle	Low flip angle (30° at 3 T). Reduces mainly T1-induced signal contamination. Decreases SNR.
Postprocessing	
Deconvolution	Model-independent or statistical approaches, not sensitive to time shift (i.e., delay) between AIF and tissue curve.
Arterial input function (AIF)	Semi-automatic or fully automatic, selecting more than one voxel in volumes containing no pathology. The AIFs should be visually assessed for correctness.
	Global AIF in case of pathologies with no substantial dispersion.
	Local AIFs in case of pathologies with dispersion, e.g., stroke or occlusion.

#### Clinical applications

**Fig. 2:**
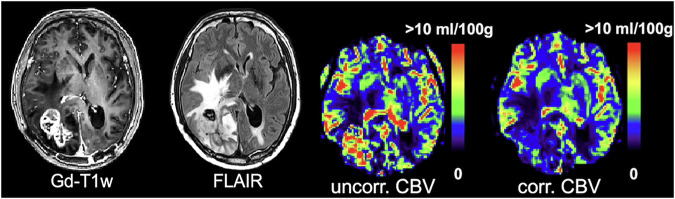
Patient with brain metastasis from anaplastic lymphoma kinase-mutated non–small-cell lung cancer. Anatomical images (Gd-T1w, FLAIR) together with uncorrected and corrected CBV. Note that leakage correction strongly reduced the elevated uncorrected CBV values in the lesion area. CBV values were normalized by setting white matter to 2 mL/100 g. Figure parts reproduced from Knutsson et al [48] under the terms of the Creative Commons Attribution License

The most common applications include characterization of brain tumor hemodynamics, for grading, treatment response monitoring and differentiation between true progression and pseudoprogression, as well as between recurrence and radiation necrosis. Another relevant application is acute stroke, where perfusion parameters contribute to identifying the ischemic penumbra, i.e., distinguishing potentially salvageable tissue from the irreversibly damaged infarct core [5]. In most cases, a T2*-weighted gradient echo (GRE) measurement is typically preferred [6]. Although T2-weighted spin-echo (SE) DSC-MRI is, in relative terms, more sensitive to capillary-sized vessels, GRE DSC-MRI provides a generally larger maximal signal drop. Since brain tumors often show a disrupted blood-brain barrier (BBB), the general DSC-MRI tracer kinetic theory, assuming an intravascular CA, becomes invalid. Leakage effects can be remedied by using preload/prebolus CA injection and/or low flip angle. Mathematical algorithms, allowing for permeability estimation, can also be applied (Fig. 2) [6, 7]. Leakage correction should preferably be employed in all diseases with BBB disruption, e.g., neurodegenerative diseases. Finally, it should be noted that, for example, in stroke and vascular occlusion, an arterial bolus dispersion may occur between the site of the measured AIF and the tissue voxel’s true AIF, which may lead to quantification errors. Local AIFs might thus be warranted in diseases where a pronounced arterial dispersion can occur [8].

#### Challenges for clinical implementation

It is imperative for successful clinical implementation that the CA bolus shape is captured and that a sufficient signal decrease is achieved. Inaccurate shape registration can be caused by slow injection rate and/or poor temporal resolution [9]. An insufficient signal decrease can occur if the TE is too short, but, on the other hand, an extensively prolonged TE can result in signal saturation, particularly in large vessels [9].

Quantification in absolute terms is generally impeded by AIF partial volume effects, differences in relaxivity between tissue and blood, and a non-linear ΔR2*-versus-concentration relationship in whole blood [6]. These methodological complications hamper the follow-up of quantitative perfusion biomarkers in individual patients as well as comparisons among individuals. However, for decisions regarding diagnosis and monitoring of treatment response, relative measurements, followed by visual assessment, are usually sufficient.

### Dynamic contrast-enhanced (DCE) MRI

#### Basics physics

**Fig. 3:**
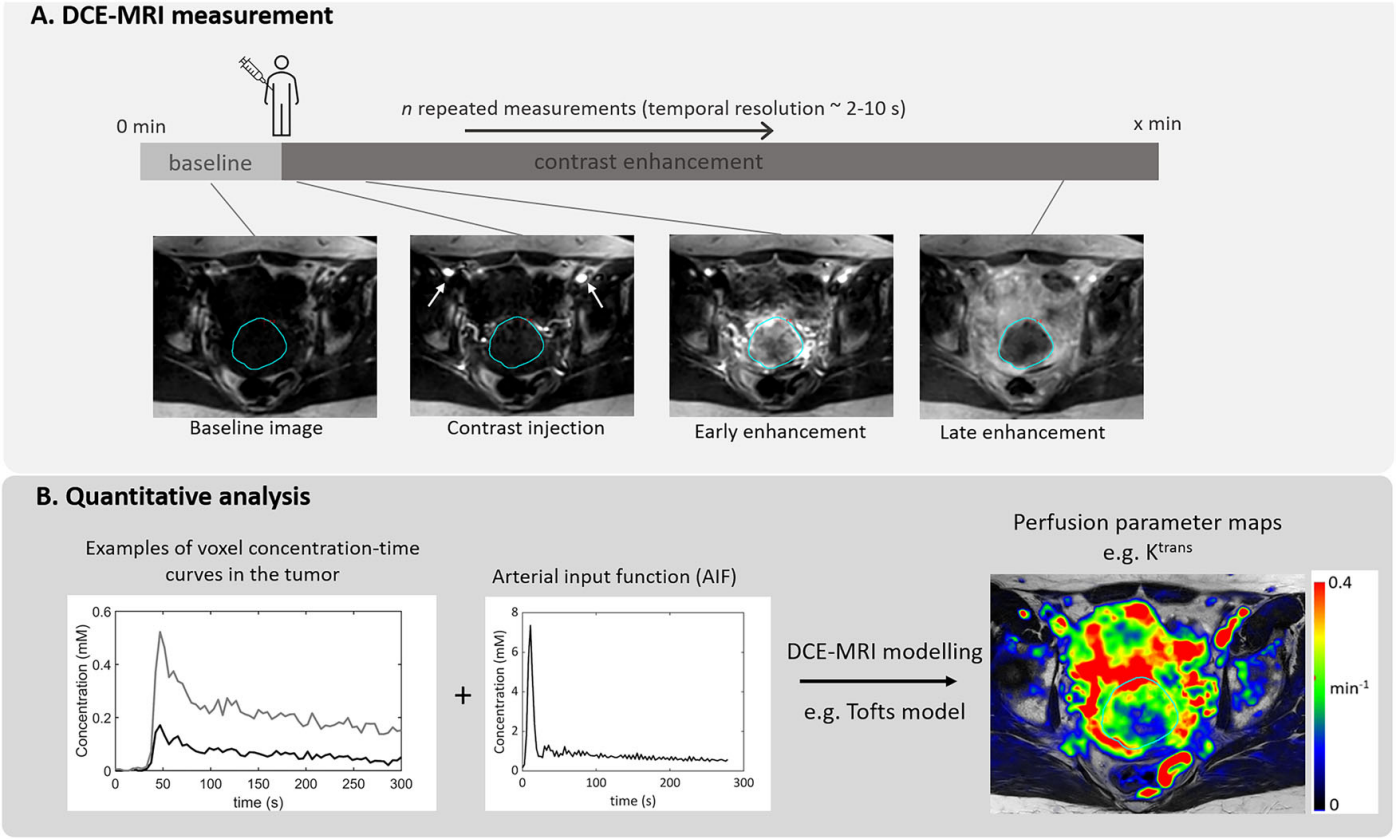
Overview of DCE-MRI methodology illustrated for a patient with cervical cancer. The measurement is shown in **A**, which typically consists of repeated measurements before, during and after contrast injection with a power injection. Example DCE images are shown from baseline, just after contrast injection, where uptake in the iliac arteries is visible, early enhancement in the tumor and the enhancement at the end of acquisition. A simplified version of the analysis is shown in **B**, where examples of concentration-time curves from the tumor region are shown together with the arterial input function (AIF). These signals are then used to extract relevant perfusion metrics. An example of the K^trans^ map is shown here as a color overlay on the T2-weighted MRI of this patient

In DCE-MRI, T1-weighted MR images are acquired dynamically before, during and after injection of a CA bolus (Fig. 3A) [10]. The images show a temporally varying signal according to CA concentration. 3D fast spoiled gradient recalled echo sequences or equivalent are recommended [11]. These are available for all vendors. The amount of T1-weighting is controlled by the repetition time (as short as possible) and flip angle (adjusted to get maximum signal, i.e., Ernst angle) [12]. Other settings vary per application, often entailing a careful trade-off between temporal and spatial resolution, spatial coverage, and signal-to-noise ratio. For perfusion measurement, a high temporal resolution (< 4 s) is essential to capture the first pass of the bolus. Permeability measurement, however, can be performed at lower temporal resolutions (10–20 s) but requires longer acquisitions (> 3 min). In recent years, acceleration techniques emerged, for instance, keyhole imaging. However, the impact on quantification is not yet fully clear and warrants further investigation. Specific recommendations for the brain, prostate, and breast are given by the QIBA DCE-MRI committee [11].

In some applications, visual data inspection or semiquantitative metrics, such as the area under the signal enhancement curves, may be sufficient for diagnostic purposes. These are faster and simpler to extract, but subjective and may not be reproducible across scans or populations. Quantitative analysis based on tracer kinetic modeling is supposed to be more objective, by giving estimates of biological characteristics, and reproducible, but it has additional requirements, which may not always be easy to implement clinically. Quantitative analysis involves multiple steps from MR signal conversion into CA concentration: AIF measurement, and fitting an appropriate pharmacokinetic model (Fig. 3B) [13]. For signal to concentration conversion, a baseline T1 map is needed (for field strengths ≥ 3 T, a B1 map is recommended to correct flip angles affected by B1 field inhomogeneities) [14]. Analysis can be performed voxel-wise or in a region-of-interest (ROI). The choice of model depends on the tissue type and data properties. The majority of applications aim to measure plasma (or blood) and extracellular-extravascular volume fractions, flow, the volume transfer constant (K^trans^), or the permeability-surface area product of the capillary wall. For a detailed explanation about these parameters and their models, we refer to other review papers [15, 16].

#### Clinical applications

In clinical care, DCE-MRI is mainly used qualitatively or semi-quantitatively, most often for distinguishing benign from malignant lesions. For example, signal-time curve assessment is included in the Breast Imaging Reporting and Data System for breast cancer screening and diagnosis [17]. In addition, visual assessment of DCE-MRI with a hepatocellular contrast agent is routinely used for the detection and characterization of liver lesions.

**Fig. 4:**
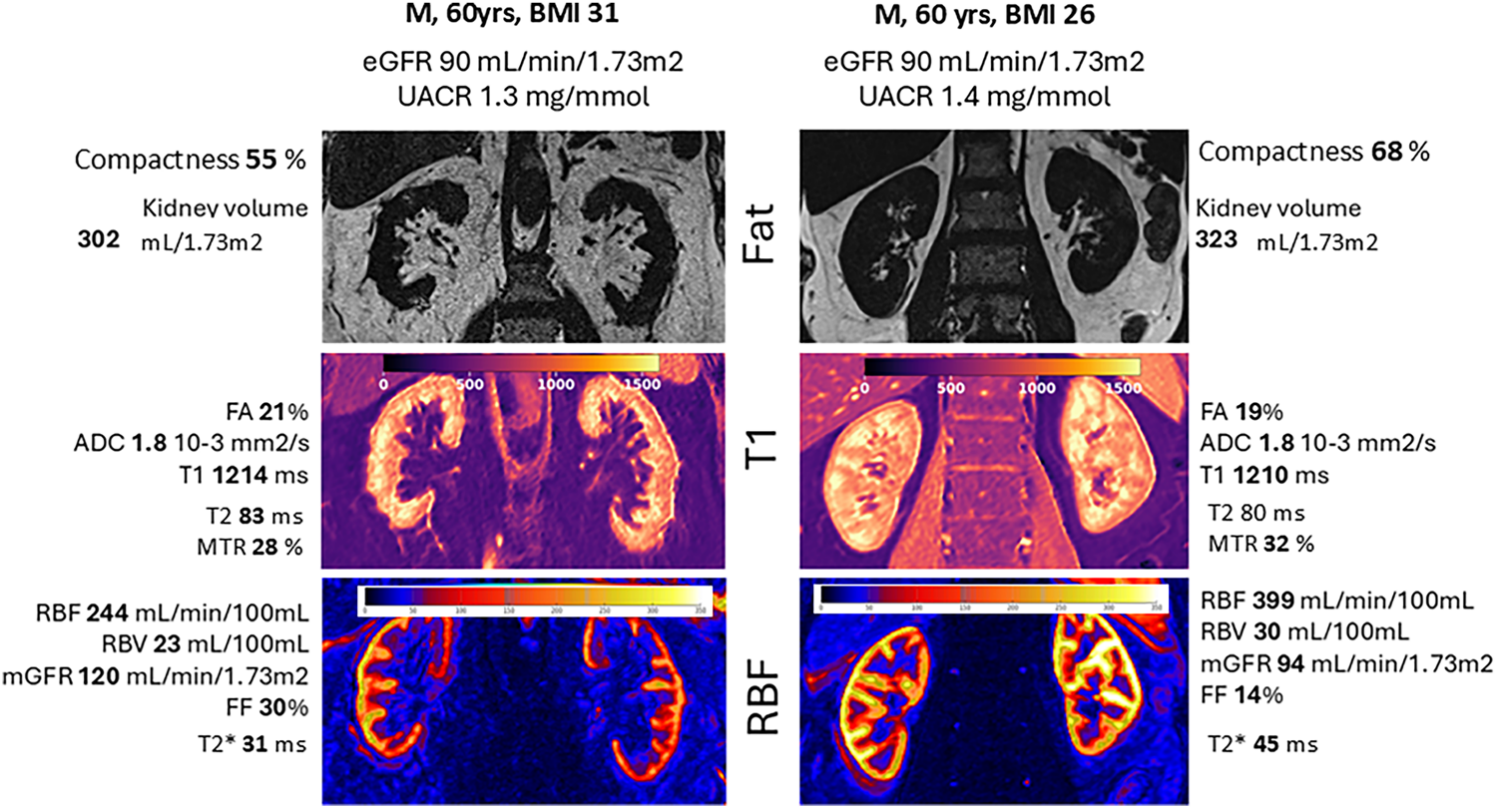
Two participants in a study on diabetic kidney disease [23], illustrating the added value of perfusion MRI in the assessment of chronic kidney disease. The subjects are demographically similar, both 60-year-old men with normal kidney function, but one has a body mass index (BMI) value in the obese range (left column), and the other falls in the overweight category (right column). The images show a DIXON fat map of their kidneys (top row), a T1 map (middle row) and a perfusion map (bottom row). Both subjects have similar kidney volume (normalized to body surface area) and similar values for structural parameters such as fractional anisotropy (FA), apparent diffusion coefficient (ADC) and T1. However, their perfusion maps show remarkable differences: the obese participant has lower perfusion but higher glomerular filtration rate (GFR)—leading to a substantially increased filtration fraction (FF) (30% vs 14%). The data suggest that while the kidneys of the overweight subject are not yet affected by underlying disease, the kidneys are in hyperfiltration—indicating they are at higher risk of kidney disease progression

In clinical research studies, DCE-MRI has shown promise as a quantitative biomarker across a wide range of pathologies [18, 19]. In the brain, DCE-MRI-based blood-brain barrier permeability has been shown to predict hemorrhage risk after stroke treatment [20]. In the more general setting of cerebrovascular disease, DCE-MRI has been applied to vascular cognitive impairment, Binswanger’s disease, dementia, small-vessel and minor stroke, type 2 diabetes, and aging [21]. DCE-MRI has also been extensively applied in oncology research for diagnosis, staging and treatment response monitoring. For example, DCE-MRI has shown prognostic value for patients with locally advanced cervical cancer treated with chemoradiotherapy, where patients with well-perfused tumors have a better prognosis [22]. In other areas, DCE-MRI has been used to characterize renal function in chronic kidney disease [23] (Fig. 4) and to assess the degree of inflammation in musculoskeletal disease [24].

#### Challenges for clinical implementation

Despite the wide range of applications in clinical research, clinical use is limited and tends to rely on visual assessment or semiquantitative metrics. Some MRI vendors and independent companies offer quantitative DCE-MRI solutions for clinical workflows, but there are currently no widely accepted indications or formal guidelines to support routine clinical use.

Clinical adoption of quantitative DCE-MRI is hindered by a lack of reproducibility. Both acquisition and analysis choices contribute to the variability of quantitative parameters, and these protocols vary between vendors, systems, and research groups [25]. This can lead to large variations, even when the same dataset is analyzed with different software [26]. Furthermore, reporting of the analysis is often incomplete, lacks standardized terminology, and is often not stored in the metadata of the output parameter maps [27].

### Arterial spin labeling (ASL) MRI

#### Basic physics

**Fig. 5:**
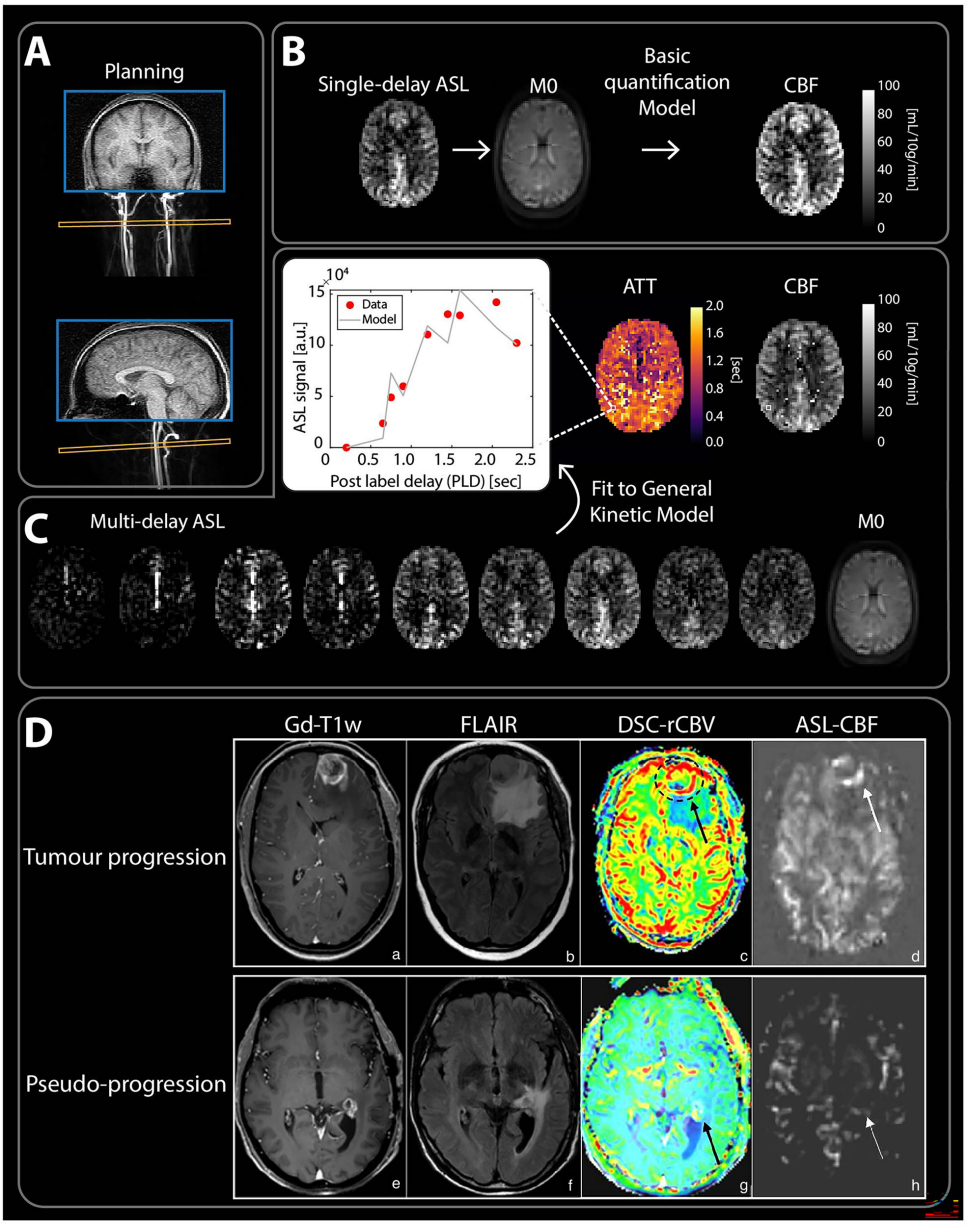
A recommended brain ASL workflow (**A**) in a healthy volunteer, including placement of the ASL labeling plane on a straight segment of the arteries. The consensus single-delay ASL (**B**), which includes an automatic quantification step with M0 map calibration to provide a (cerebral blood flow) CBF map on the scanner, and a multi-delay ASL (**C**) image sequence providing CBF and arterial transit time (ATT) maps using a voxel-wise fitting algorithm to Buxton’s general kinetic model. **D** Clinical examples of post-contrast T1w images (**a**, **e**), T2 FLAIR images (**b**, **f**), DSC-rCBV maps (**c**, **g**) and ASL-CBF maps (**d**, **h**) for a patient with confirmed tumor progression (top row) and a patient with pseudoprogression (bottom row). A peripheral enhancing rim with increased CBV and CBF is associated with suspicion of tumor progression, while a peripheral enhancing rim with low or normal CBV and CBF is associated with pseudoprogression (reproduced from van Dorth et al [33] under the terms of Creative Commons CC-BY-NC license)

ASL-MRI uses blood-water spins to produce a safe, endogenous, non-contrast tracer. This credits ASL with being specific (ASL-MRI signal originates only from labeled blood) and completely non-invasive. It is therefore an ideal choice when contrast agents are contraindicated. Labeling is performed (Fig. 5A) by preparing the longitudinal magnetization (either by saturation or inversion) of flowing spins in the brain-feeding arteries. The label will decay with the local T1 relaxation (1.6 s in blood); hence, for a readout occurring 3 s after labeling, only 16% of the magnetization remains. Therefore, the basic dilemma to achieve adequate SNR is to balance the time needed for spins to reach tissue (depending on blood velocity) while avoiding too much signal loss due to T1 decay. This also implies that T1-shortening contrast agents should not be administered prior to ASL-MRI. Finally, as most labeled spins cross the BBB and accumulate in brain tissue, CBF can be approximated by the amount of spins detected in tissue [28] (Fig. 5B, C).

For brain imaging, pseudocontinuous labeling should be used with a labeling duration and post-labeling delay of 1800 ms, background suppression, and segmented 3D readout under 300 ms. If hemodynamic impairment is expected, the delay should be increased to 2000–2400 ms, and a longer labeling duration should also be considered. For neonates, a prolonged post-labeling delay (2000 ms) is recommended. Due to ASL-MRI’s low SNR, the spatial resolution should not be too high (typical voxel size is 3 × 3 × 5 mm³).

#### Clinical applications

The three main MRI vendors offer a single-delay background-suppressed 3D pseudocontinuous ASL-MRI sequence with M0 calibration for CBF quantification following the 2015 consensus recommendations [28], providing CBF maps as direct output. Typical artifacts in single-delay ASL-MRI can confound CBF quantification yet still provide important diagnostic information: a hyperintense arterial signal (so-called arterial transit artifact [29]) indicates delayed arrival (e.g., arterial stenoses) [30]; and a hyperintense venous signal can localize draining veins or the nidus of arteriovenous shunts [31]. CBF quantification is not always necessary for diagnosis; a description of hyper-/hypoperfusion is often sufficient [32]. ASL-MRI is indicated for diagnosing and grading brain tumors, mostly showing increased CBF in glioma, and for differentiating progression from pseudoprogression post-treatment (Fig. 5D) [33]. Perfusion imaging, including ASL-MRI, is recommended for managing Moyamoya disease [34].

Recently, the research community recommended specific implementations for multi-delay ASL-MRI [35], which improves CBF sensitivity and provides additional timing information. However, not all vendors currently offer multi-delay ASL-MRI with automated quantification. Whereas the brain currently is the main organ in which ASL-MRI is applied, body applications are also gaining popularity [36].

#### Challenges for clinical implementation

Single-delay ASL-MRI was successfully implemented a decade ago owing to close collaboration between the research community, clinicians, and vendors. However, clinical adoption still faces challenges that multi-delay ASL-MRI does not fully address, including SNR, labeling plane positioning, proper shimming at the labeling region, selecting the appropriate post-labeling delay (PLD), and clinicians’ awareness. Another obstacle is the contention that a CBV map is essential for tumor evaluation, despite similar information (i.e., CBF) being obtainable non-invasively with ASL-MRI.

### Intravoxel incoherent motion (IVIM) MRI

#### Basic physics

IVIM-MRI extracts microvascular perfusion information from multi-b-value diffusion-weighted MRI [37]. The method uses a bi-exponential signal equation to model the signal arising from both the motion of blood in the microvascular bed at low b-values and from molecular diffusion effects at high b-values.

**Fig. 6:**
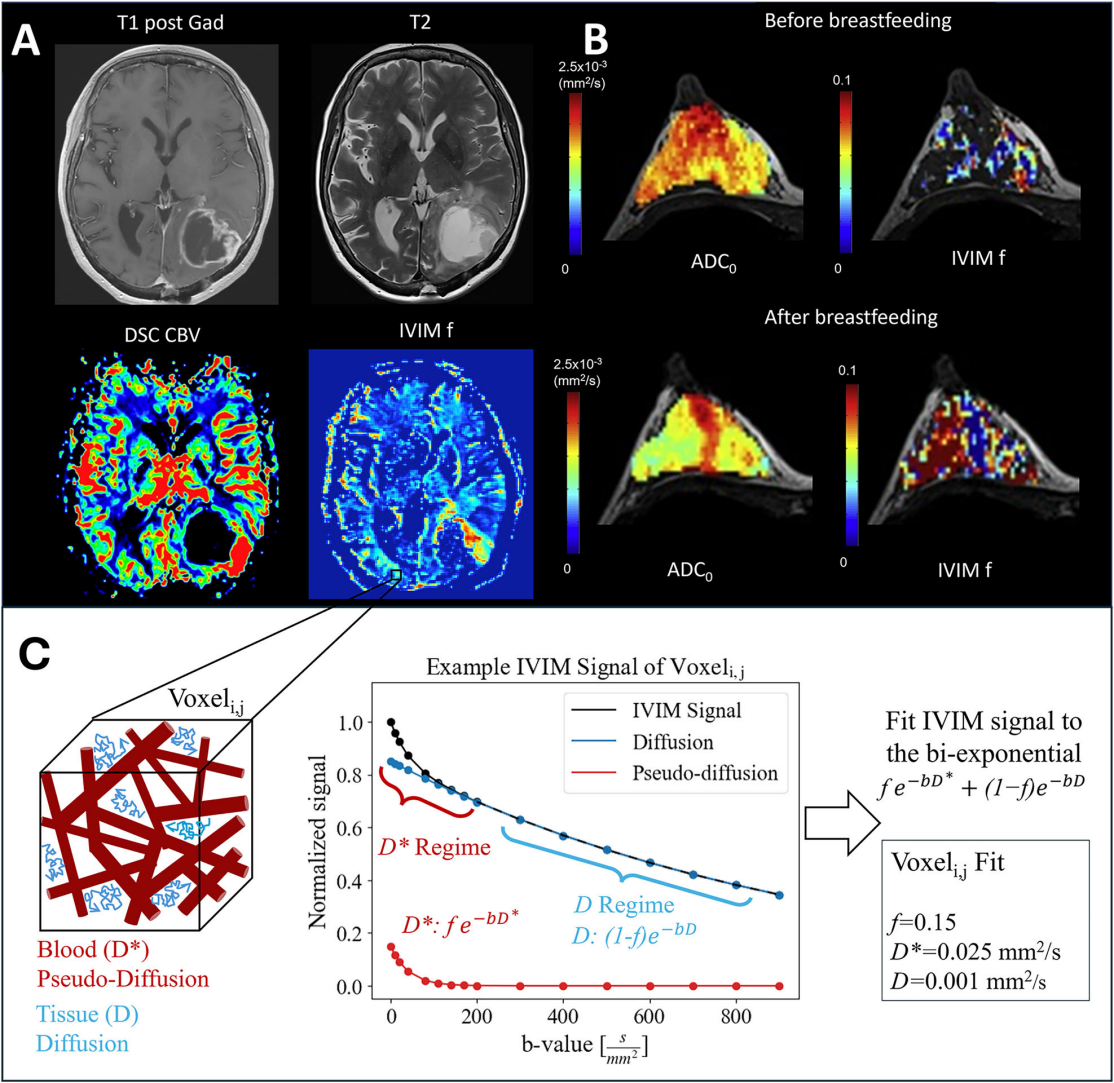
High-grade glioma (**A**), with a clear increase in both DSC CBV and intravoxel incoherent motion (IVIM) perfusion fraction (f). Note that the increase in CBV and f is not exactly at the same location; while both are perfusion parameters, they are different methods and not expected to correlate perfectly. **B** ADC0 (ADC calculated between b-values of 200 and 2500 s/mm^2^) and IVIM f maps before and after breastfeeding in a 32-year-old lactating volunteer with a 7-month-old baby, showing a clear increase in IVIM perfusion after breastfeeding. Modified with permission under the Under a Creative Commons license from Iima et al [38]. **C** Example of the IVIM signal equation model with 16 b-values, perfusion fraction f = 0.15, pseudo-diffusion coefficient D* = 0.025 mm^2^/s, and diffusion coefficient D = 0.001 mm^2^/s. Commonly, b-value sets are done in at least 3 directions and include at least one b-value from the pseudo-diffusion regime (0–200 s/mm^2^) and one from the diffusion regime (200–1000 s/mm^2^) in addition to 0, 200, and 800 s/mm^2^. The b-value sets used in literature vary based on organ and disease of interest, and the most common method of fitting the signal is a segmented fit. This is done by fitting D and f using only b-values in the diffusion regime and then fitting D* of the bi-exponential, using all b-values, after fixing D and f from the first step

The bi-exponential IVIM signal model is fit to the measured signal at multiple b-values to extract a perfusion-free diffusion coefficient D, a perfusion-related ‘pseudo-diffusion’ coefficient D*, and the fraction of signal arising from the perfusion compartment called the perfusion fraction f (Fig. 6C). The perfusion fraction, f, is correlated to blood volume; D* is related to mean transit time; and the scalar product, fD*, is related to blood flow. Table 2 summarizes general protocol recommendations, including adaptations to different organs, with further technical recommendations provided by the ISMRM IVIM Workshop Consensus.

**Table 2:** Recommended protocol for IVIM-MRI measurements

Parameter	Recommendation
Experimental setup	
Field strength	1.5 T or 3 T; preference for 3 T for improved SNR
Pulse sequence	2D single-shot spin-echo (SE)
Readout	EPI
TE	Minimum
TR	3–5 s
Slice thickness	1.2–5 mm
In-plane resolution	1–4 mm
Parallel imaging factor	2
Gradient directions	3 or more
Averages	1, benefit of more averages at high b-values or regions with low SNR
Diffusion gradients	Monopolar or bipolar
Minimum number of b-values	0, 200, +1 upper limit b (500–1000) s/mm^2^, depending on organ
Additional b-values	Add additional b-values evenly in the [0, 200] and the [200, b_max] intervals
Slice orientation	Axial
Fat suppression	SPAIR (+SSGR if available) for breast, muscle, abdomen; SPIR for brain
Postprocessing	
Bi-exponential $f{e}^{-b{D}^{* }}+(1-f){e}^{-{bD}}$	Segmented fitting: (1) signal normalized to b = 0 s/mm^2^ is fit to a mono-exponential for only high b > 200 s/mm^2^ and used to extract f and D. (2) D* is estimated by fitting the full IVIM equation to all b-values with D and f fixed from step 1.

#### Clinical applications

IVIM offers three key advantages:

1. Obtains microcirculation information without the need for contrast injection.
2. Essentially, local measurement, independent of blood flow path or delay, as both excitation and read-out are performed in the same plane.
  This is particularly valuable in cases of slow blood flow, such as stroke or severe artery stenosis.
  Local blood flow might be conserved through appropriate collaterals.
3. Provides complementary perfusion information to other methods due to its fundamentally different methodology.

IVIM-MRI can be clinically used in all perfusion paradigms (Fig. 6) [38]. For example, perfusion fraction f can be used to differentiate high-grade from low-grade brain glioma [39] to distinguish malignant from benign breast lesions [40], or to differentiate head and neck lymphoma from squamous cell carcinoma and malignant salivary gland tumor [41]. Meta-analyses have demonstrated f and D in the abdomen as a method to differentiate and grade pancreatic, hepatocellular, renal, cervical, prostate, and liver lesions [42]. Beyond oncology applications, IVIM-MRI can be used in stroke to measure the decrease in perfusion in the infarct core as well as the quality of the collateral blood flow in the penumbra [43, 44], while renal IVIM has been shown to fit better than mono-exponential or non-Gaussian diffusion and demonstrated the ability to detect chronic kidney disease and acute graft dysfunction [45].

#### Challenges for clinical implementation

Key challenges for clinical implementation include poor repeatability/reproducibility of IVIM-MRI parameters (especially D*), extended scan time, motion, lack of standardization in acquisition and analysis regarding diffusion directions, averages for improved SNR, differing b-value sets depending on organ of interest and absence of widely accepted clinical guidelines. Efforts are ongoing to address these challenges.

### Road to clinical implementation

As shown, perfusion MRI could be relevant for a multitude of applications. However, clinical adoption varies across techniques and all face challenges, especially when it comes to quantification. Currently, the acquisition protocols are available on all scanners and can be tailored to the specific application with the institutional application specialist, but most are not yet standardized. Moreover, postprocessing is not directly available at the scanner for all techniques. This typically requires third-party software tools (commercial, in-house developed, or open-source) [46, 47]. Improving reproducibility of perfusion MRI is an active area of research by several communities, among others Quantitative Medical Imaging Coalition (QIMC, formerly QIBA), with a focus on data acquisition, and the ISMRM Open Science Initiative for Perfusion Imaging (OSIPI), focusing on data analysis.

Steps to be taken toward further clinical implementation, depending on the respective technique, include:

- Improve accessibility in collaboration with vendors and third parties to ensure standardized acquisition protocols and postprocessing directly on the scanner.
- Multicenter trials to establish reproducibility for technical validation.
- Raise awareness and expand knowledge among clinicians in collaboration with clinical societies and journals, for instance, clinical users sharing their experience.
- For clinical validation, robust clinically actionable thresholds need to be established through multicenter clinical trials.

## Summary statement

Perfusion MRI techniques—including DSC-MRI, DCE-MRI, ASL-MRI and IVIM-MRI—hold significant potential as imaging biomarkers across various clinical applications. However, the level of clinical adoption, particularly for quantitative parameters, varies per technique. A key barrier to broader clinical translation is the lack of standardization in acquisition and analysis protocols, which leads to poor reproducibility and limits clinical confidence. Additionally, some of these techniques remain underutilized due to limited awareness and understanding among radiologists.

**Fig. 7:**
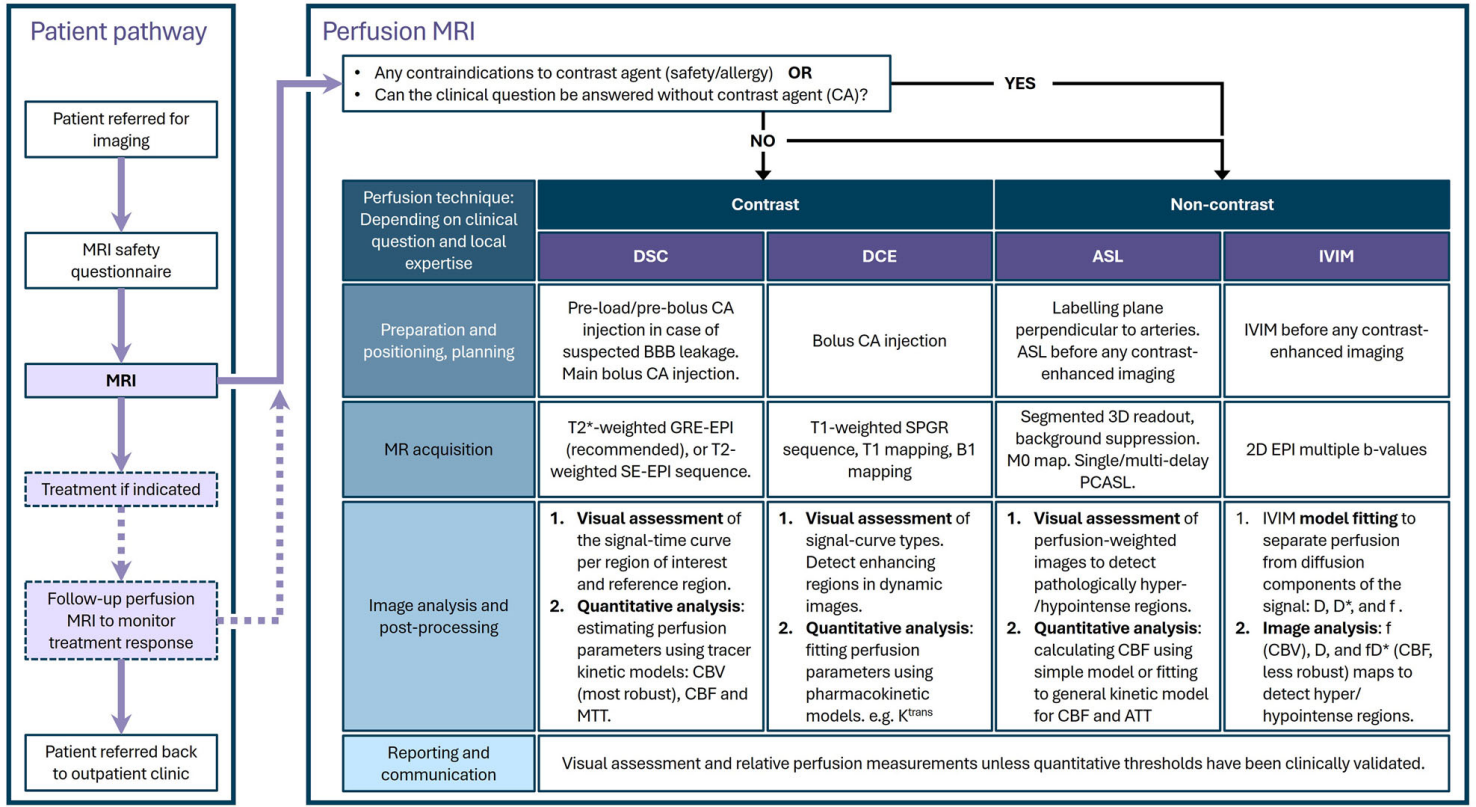
Flowchart of the imaging pathway of perfusion MRI

These practice recommendations aim to expand radiologists’ knowledge of perfusion MRI techniques and provide practical guidance on acquisition and analysis, including a flowchart covering perfusion MRI imaging pathways (Fig. 7). The goal is to support the integration of perfusion MRI into routine clinical workflows. Further supporting this effort, initiatives such as the Quantitative Medical Imaging Coalition (former QIBA) and the ISMRM Open Science Initiative of Perfusion Imaging (OSIPI) are developing standards and guidelines. Ultimately, the successful integration of state-of-the-art perfusion MRI into clinical practice requires close collaboration among clinicians, researchers, and industry partners.

## Patient summary

Perfusion ensures the constant delivery of blood and nutrients to organs. Both too-low (stroke) and too-high perfusion (cancer) can be signs of disease. There are four main perfusion magnetic resonance imaging techniques. Some of them require the injection of a contrast agent, while others do not. Which perfusion technique to use depends on the organ and disease. Perfusion MRI provides critical information for diagnosing, staging, and management of disease, but not all of them are regularly used in the clinic. To improve clinical use, clinicians, researchers and MRI vendors need to work together to develop guidelines.
